# Yes we can! Improving medical screening for intimate partner violence through self-efficacy

**DOI:** 10.5249/jivr.v3i1.62

**Published:** 2011-01

**Authors:** John R. Chapin, Grace Coleman, Erin Varner

**Affiliations:** ^*a*^Pennsylvania State University, USA.; ^*b*^Crisis Center North, USA.

## Abstract

**Background:**

Because individual practitioner's commitment to routine screening for IPV is the greatest predictor that women will be screened and referred for services, it is vital that screeners are dedicated, knowledgeable, and confident in their ability to recognize and assist victims of violence. Self-efficacy has been consistently linked in the literature with successful outcomes.

**Objectives:**

Intimate partner violence (IPV) constitutes a major public health problem. In the absence of Federal or State regulation, individual hospitals and systems are left to develop their own policies and procedures. This paper describes the policies and procedures developed by an American domestic violence counseling and resource center.

**Design:** Post test surveys were used.

**Settings:** Hospitals, medical offices, and medical schools surrounding an urban area in Pennsylvania participated.

**Participants:** 320 nurses and medical students participated in training provided by a domestic violence center.

**Methods:**

Post test surveys measured self-efficacy, the perceived usefulness of screening the accessibility of victim services, understanding of obstacles faced by victims, and knowledge-level regarding local IPV services. Participants also self-reported their gender, age, race, and position with the hospital system.

**Results:**

Nurses and medical interns exhibit a wide range of self-efficacy regarding their ability to screen victims of intimate partner violence. Intimate partner violence (IPV) training yielded participants who were better informed about IPV services and the obstacles faced by victims.

**Conclusions:**

In the absence of uniform screening guidelines, hospitals, systems, and individual practitioners must be vigilant in screening procedures. Partnerships with women's centers may provide valuable resources and training that may ultimately improve patient care.

## Introduction

Intimate partner violence (IPV) constitutes a major public health problem. IPV, also known as domestic violence, domestic abuse, or spousal abuse can be broadly defined as a pattern of abusive behaviors by one or both partners in an intimate relationship such as marriage, dating, family, friends or cohabitation. Trauma associated with IPV accounts for two to four percent of all women seen in hospital emergency departments in the United States, and five to 15 percent of women seen in hospitals have a recent history of domestic violence. While the majority of injuries sustained by IPV victims are classified as superficial contusion, abrasions, and lacerations, an estimated 73,000 hospitalizations and 1,500 deaths among women are attributed to IPV annually.^1^

Medical professionals, victim advocacy groups, and other organizations have been working together to improve the health care response to victims of IPV. Hospitals and health care systems are designing and implementing domestic violence training, screening, and intervention programs across the United States. Despite the prevalence of IPV, most women are not asked about their safety during an annual exam or visit to the hospital: Fewer than 10% of primary care physicians routinely screen for IPV during regular office visits. Similar findings have been reported for other healthcare settings.^2^

According to the Family Violence Prevention Fund,^3^routine face-to-face screenings by skilled healthcare providers markedly increase the identification of IPV victims. Individual practitioner's commitment to routine screening for IPV is the greatest predictor that women will be screened and referred for services. Following a brief review of the self-efficacy literature, the current paper describes a medical screening program designed to improve screening effectiveness.

**Self-Efficacy**

Because individual practitioner's commitment to routine screening for IPV is the greatest predictor that women will be screened and referred for services, ^4^it is vital that screeners are dedicated, knowledgeable, and confident in their ability to recognize and assist victims of violence.

Self-efficacy^5^is the conviction that one can successfully execute the behavior needed to produce a desired outcome. For example, Nathanson, Eveland, Park and Paul^6^found that mothers who believed they could make a difference in their children’s lives by limiting their exposure to violent television were likely to actively monitor TV viewing. Mothers who believed they couldn’t make a difference monitored less or not at all. A number of studies examine self-efficacy within the health-care context. Maly, Liu, Leake, Thind, and Diamant^7^studied 921 low-income women with breast cancer in the United States. Greater patient-perceived self-efficacy regarding communication with physicians predicted pain resolution among patients. While physician awareness was also a significant determinant of depression resolution, patient self-efficacy played a more important role in nausea resolution. The authors conclude that physicians under-recognized depression among breast cancer patients, but appropriate patient–physician communication can increase patient self-efficacy, resulting in qualitative differences in patient care.  Tousman, Zeitz, and Taylor^8^studied 21 adult asthma patients in a self-management program in the United States. Small groups met for seven weeks. Intervention techniques included interactive discussions, problem solving, social support, and a behavior modification procedure. Results included improvements in self-efficacy, which were related to patient depression and to overall quality of life. Patients exhibiting higher self-efficacy self-reported better compliance behaviors, including self-monitoring and daily exercise.

Given the potential impact of self-efficacy on the effectiveness of health-care providers, the following research questions are posited:

RQ1: How confident are medical personnel in their abilities to screen patients for intimate partner violence?

RQ2: What factors influence the self-efficacy of medical personnel regarding effective patient screening?

Medical Screening for IPV: Policies and Procedures

In the absence of Federal or State regulation, individual hospitals and systems are left to develop their own policies and procedures. This paper describes the policies and procedures developed by Crisis Center North (CCN), a domestic violence counseling and educational resource center providing services in Allegheny County, Pennsylvania, in conjunction with a local hospital system and the local District Attorney’s office. Over 3,000 Protection From Abuse (PFA) orders were filed in Allegheny County in 2007.^9^The CCN Medical Advocacy Program began in 1999 by developing medical protocols and procedures that would positively identify patients and victims of IPV and then provide healthcare workers with access to a trained medical advocate. Given the growing literature on self-efficacy in health–care settings, the CCN medical advocacy program sought to measure self-efficacy and to explore factors that may impact self-efficacy among medical personnel regarding screening for intimate partner violence.

CCN endorses the following guiding principles that are followed when dealing with potential or actual IPV victims:

The safety of the victim and any children is a priority. Respecting the integrity and authority of each battered individual to make his/her own life choices is paramount. 

The program recognizes that perpetrators are responsible for their abusive relationships and for stopping the behaviors. Intervention and resources are available in the community and within health care systems for helping perpetrators of abuse.

Advocacy on behalf of the victim is the cornerstone of the program.

At first contact with a potential IPV victim, an assessment is conducted by professional staff. Professional staff includes, but is not limited to, RNs, CRNPs, PAs, social workers, clinical psychologists, and physicians. If threatening behavior is demonstrated by an accompanying perpetrator, security is notified. If the injury involves stabbing, gunshot wound, or assault with a deadly weapon, law enforcement and security are notified. Law enforcement officers are also notified if the victim desires to press charges regardless of the extent of the injuries.

Upon admission to the ED, professional staff will initiate/complete the IPV screening process on all patients, 14 years of age or older, in a private setting. In an empathic, nonjudgmental manner, staff frames the screening question with the statement: “Because violence is common in people’s lives, I have routinely started to ask all my patients about it.” Subsequent questions, which are asked, are:

(1)“Are you emotionally and physically safe with the person (partner) you are with?”

(2)“Are you safe in your home?” (If partner lives with her/him.)

(3)“We can help you. Do you want help today?”

If the individual answers “yes” to the last screening question, a medical advocate is consulted. If the patient denies violence/abuse and does not have conflicting indicators, a negative screen is documented. If the patient denies violence/abuse and does have conflicting indicators, (such as the type of injury presented by the patient may not be congruent with the injuries seen), staff redirect the question to the patient as: “Many times, when I see patients with injuries like yours, it means that someone has tried to hurt them. Is this happening to you?”

Professional staff documents these actions in the medical record. The screening process is documented in the patient’s medical file. The medical provider documents the patient’s response to the screening question on the nursing assessment form. In addition, staff documents, whether or not signs of abuse and neglect are observed.

Confidential screening is completed in a private environment. If the alleged abuser wishes to stay with the potential victims, he/she is directed to wait in the visitors’ lounge “until the assessment is completed by the doctor/nurse.” At no time is a patient screened about abuse in the presence of others.

Hospital staff members complete documentation, which should be clear and objective.Documentation should include:

Date and time of injury;

Nature/cause of injury using the victim’s own words (i.e. patient states that “xxx”;

Avoid long descriptions (i.e. patient describes in detail the argument that lead to the injury) which deviate from the medical problem; this information is inadmissible and may be counter-productive if inconsistent with court testimony;

Any witnesses to the assault/”accident”;

A description of wounds (color, location, size, etc.);

Photographs of injuries;

Notations of any other evidence of abuse such as torn clothing and jewelry; emotional state (i.e.: anxious, calm, and withdrawn);

Name and relationship of person accompanying the patient;

Name, badge number, and phone number of law enforcement officer accompanying the victim.

Medical providers include detailed descriptions of the nature and location of all injuries, new and old. Body charts and injury maps are utilized to document the injuries. Patients are asked about the cause of old injuries. Medical providers record any pattern of injuries that they find, such as pattern contusions consistent with human bite marks. If it is suspected that the patient has given a false explanation as to the cause of the injuries, providers document the inconsistency of the individual’s statement in relation to the injury observed. Providers are encouraged to write such statements as, “These injuries do not appear to be consistent with bumping into a door, but are consistent with blunt-force trauma to the head.”

## Methods

**Participants**

The target group for Crisis Center North training is nurses, interns (medical students), and administrators who typically involved in triage or patient screening. Previous (unpublished) findings in the region suggest that medical students/interns are more open to IPV screening than more experienced staff. The survey group consisted of medical students (76%), nurses (22%) and administrators (2%) from a large hospital system in Pennsylvania (N= 320). The sample was 70% female, 87% Euro-American, and ranged in age from 19 to 61 (M= 29, SD=11.1). The sessions were part of on-going training required of personnel of a large hospital system. Sessions were scheduled throughout a single calendar year.

**Materials**

Self-efficacy was measured by the following item: “I feel confident in my ability to screen patients for intimate partner violence.” Responses were on a Likert-type scale ranging from 1 (not at all) to 7 (extremely confident). Similar 7-point scales were also used to measure the remaining variables. Perceived usefulness of screening was measured by the following item:  “Patient screening is a useful tool in identifying victims of intimate partner violence” (1 = Not at all; 7 = extremely useful). The accessibility of victim services was measured with the following item: “IPV services available to patients at the hospital are easily accessible” (1 = strongly disagree; 7 = strongly agree). Understanding of obstacles was measured with the following item: “I have a good understanding of obstacles which impact a victim’s ability to leave his or her situation” (1 = strongly disagree; 7 = strongly agree). Knowledge-level regarding IPV services was measured by the following item: “I feel well informed about the services available to victims of intimate partner violence” (1 = strongly disagree; 7 = strongly agree). Participants also self-reported their gender, age, race, and position with the hospital system.

**Procedures**

Hospital personnel within the system are required to participate in training each year for professional development. Personnel are also tested on their knowledge of multiple contemporary issues, including IPV. IPV training provided by Crisis Center North was among the options offered. Participants completed a post-test immediately following the one-day session. In addition to the study variables, post-tests included evaluative items, such as quality of the presenter and newness of the information.

## Results

Research Question 1 asked how confident medical personnel are in their ability to screen patients for intimate partner violence.Table 1displays summary statistics for study variables including self-efficacy. Responses ranged from 1 (not at all) (1.9%) to 7 (extremely confident) (8.2%), with an average of 5.1 (confident).

Research Question 2 asked about the factors influencing the self-efficacy of medical personnel regarding effective patient screening. Results indicate that self-efficacy did not vary significantly by age, gender, or position at the hospital.Table 2displays zero-order correlations among the variables predicting self-efficacy. Standard multiple regression was used to identify the predictors of self-efficacy.Table 3displays the regression analysis.

Knowledge of available services was most strongly related to self-efficacy within the regression model. Participants ranged from 3 (somewhat informed) (.7%) to 7 (extremely informed) (27.8%), with an average score of 5.8 (well informed). As knowledge of services increased, self-efficacy also increased. The second strongest relationship was between self-efficacy and obstacles (I have a good understanding of the obstacles which impact a victim’s ability to leave their situation). Participants ranged from 1 (not at all) (.7%) to 7 (very good) (42.1%), with an average score of 6.2. As the understanding of obstacles increased, self-efficacy also increased. Beliefs about easy access to services was a significant correlate of self-efficacy, but failed to explain any unique variance in the regression model once shared variance was controlled for. Ironically, self-efficacy was not predicted by whether or not hospital personnel believed their current screening tools were useful to identify IPV victims.

**Table:** Table 1. **Summary statistics for study variables**

	Mean	(SD)	Range
Self-Efficacy	5.1	(1.2)	1-7
Knowledge	5.8	(0.5)	1-7
Obstacles	6.2	(0.9)	1-7
Access	5.7	(1.0)	1-7
Useful	6.0	(0.9)	1-7

**Table:** Table 2.**Zero order correlations among variables predicting self-efficacy**

	2	3	4	5
1.Self-efficacy	.48**	.42**	.25**	.03
2.Knowledge of services	---	.48**	.41**	.01
3.Obstacles		---	.35**	.23**
4.Access			---	.10
5.Useful				---

Note. **p< .01.

**Table:** Table 3.**Summary of Linear Regression Analysis for Variables Predicting Self-efficacy**

			Adj.r2 = .26/N = 298
Predictor	B	SE B	β
Knowledge of services	.43	.10	.35***
Obstacles	.31	.10	.24**
Access	.03	.10	.02

**p<.01.***p<.001

## Discussion

Self-efficacy has been consistently shown to predict successful goal attainment. Results from a survey of emergency medical personnel shows that nurses and medical interns exhibit a wide range of self-efficacy regarding their ability to screen victims of intimate partner violence (IPV). Since Federal legislation is not yet in place in the United States, individual states, systems, hospitals, and advocacy groups are left to determine their own policies and procedures. The role of the community provider is paramount to providing quality patient care to IPV victims. IPV training provided by a domestic violence center yielded participants who were better informed about IPV services and the obstacles faced by victims. Knowledge of services and obstacles, in turn, were related to self-efficacy. The results suggest that current trends in national legislation could have a positive impact on patient care. In the absence of a national standard, the partnerships between advocacy groups and hospitals emerging throughout the United States are likely increasing the proper identification of IPV victims screened in medical centers for related injuries, as well as providing potentially life-saving referrals to services.

A number of limitations should be noted. The study is based on a convenience sample of medical professionals gathered for IPV training. Individuals who participated in the training are likely to differ from those who chose a different topic to meet their training obligation. The preference for the topic may have resulted in a bias among participants and possibly variance in self-efficacy.  It is also problematic that the measures were taken at the end of training. While training may have influenced responses, it was important to gauge self-efficacy after medical professionals were more knowledgeable about intimate partner violence. A pre-post design should be used for future studies. The study variables consisted of single-item measures.

Results should be considered preliminary, but may provide useful information for practitioners and for subsequent research. A long literature establishes that self-efficacy predicts goal achievement. The literature has only just begun establishing direct applications to the medical field; however, applying established results suggests that medical personnel who are confident about their ability to accurately screen IPV victims will be more successful in the screening process. In the current study, knowledge of services available to victims of intimate partner violence and obstacles faced by victims were both related to self-efficacy. Partnerships between hospital systems or medical schools and non-profit women’s centers may provide cost-effective cross-training, as well as provide potentially life-saving services to victims.
